# Linking Place and Mind: Localness As a Factor in Socio-Cognitive Salience

**DOI:** 10.3389/fpsyg.2016.01143

**Published:** 2016-07-29

**Authors:** Marie M. Jensen

**Affiliations:** Department of Culture and Global Studies, Aalborg UniversityAalborg, Denmark

**Keywords:** Tyneside English, language variation, social value, socio-cognitive salience, exemplar theory, enregisterment

## Abstract

This paper investigates the salience of vernacular Tyneside forms on the basis of theories of enregisterment and exemplar processing. On one level, exemplar theory provides a psycholinguistic account of how the link between social value and linguistic features is possible. Conversely, integrating the notion of social value into exemplar theory extends the value of this originally cognitive theory to social domains. It is suggested that the association of social value and particular local, linguistic forms may contribute to the salience of these forms among local speakers. The empirical work reported here takes the form of a questionnaire study, which aims to uncover Tyneside inhabitants' awareness of forms as well as their affiliation with the local community. Results showed differences in frequency perceptions between participants themselves and others which indicate that speakers can identify local forms as such, but that the variety is stigmatized. The strength of local affiliation correlated with participants' own language use and it is suggested that this can be accounted for by employing a social personae explanation, where speakers use certain salient forms to index local belonging despite overt stigma.

## Introduction

Within sociolinguistics, studies of the meaning of place (often in local or regional terms) to speakers' language use and identity are many. Place is seen as a natural external variable in sociolinguistic studies, mainly because research in this field has always been engaged in the study of language variation across different localities (early dialectology being a prime example). In addition, sociolinguistic studies have investigated the meaning of places as a factor which shape speakers' linguistic identities, their sense of self and, importantly, their self-representation. Borrowings from linguistic anthropology have further enriched the area of study, most recently with the terms indexicality (Silverstein, 2003) and enregisterment (Agha, 2003) becoming commonplace in sociolinguistic studies (Johnstone et al., 2006; Johnstone and Kiesling, 2008; Beal, 2009; Johnstone, 2009, 2010).

While these two terms, which account for processes at play on the social level, work well in underpinning sociolinguistic patterns of variation and change, especially when these are concerned with matters of identity, as such they do not present ideas which have not already been posited by earlier sociolinguists (such as Labov, 1972). In addition, what they ultimately aim to capture can be summed up by the term *salience*; a term which has many uses and connotations in many fields and which is not, in itself, easily accounted for. Finally, what is perhaps lacking is an account of how these processes can operate from a cognitive perspective. How can we support these ideas of locality having an impact on speakers' language use through arguments about their identity and not as mere reflections of variation due to differences in locality, e.g., Manchester vs. Liverpool?

This article presents a sociolinguistic study of the role of local attachment by Tyneside English speakers in their awareness and perceptions of local forms' frequency of use and local status.

The data was collected via questionnaires which asked participants to rate example sentences with regards to their frequency of use. In addition, participants were also tested on their ability to identify local forms and they were assessed with regards to their local affiliation. Five variables were included in the study: (do+NEG), (our), (told), (throw), and (go). In the interpretation of results, I will suggest that the perception of the forms as unique to Tyneside (and thus encapsulating localness) makes them occupy an especially salient position in speakers' minds (see Honeybone and Watson, 2013 for a similar argument for phonological forms in Liverpool English based on an analysis of contemporary dialect literature). We can find support for this suggestion in exemplar theory, if we view language as a complex adaptive system (CAS), where social and cognitive factors both play equal roles in the shaping of language use, both on the individual and on the community level (Beckner et al., 2009; Bybee, 2010).

First, I set up the theoretical underpinnings for the study of local vernacular forms in Tyneside English presented here and briefly introduce the topic of salience from a sociolinguistic perspective and link it to indexicality and enregisterment. I then place the sociolinguistic approach to salience within an exemplar theoretical framework (and a wider conceptualization of language as a CAS) in order to show how the sociolinguistic approach can be supported from a psycholinguistic point of view. In the third section, I introduce the questionnaire study, which forms the empirical basis for this paper, and briefly account for the five vernacular variables under study. The data is then analyzed quantitatively and, in section four, I discuss the results in relation to salience and suggest the concept of *social personae* as a way to account for the patterning found.

## Salience in sociolinguistics

While the topic of salience is hardly new, finding common ground between the many publications on this topic can be difficult as many approach the topic from vastly different perspectives. Within sociolinguistics, the early work of Labov (1972, 1994) and Trudgill (1986) seems to form the basis on which definitions and later studies of salience have been based. Both Labov and Trudgill take as their focus the speech community as a whole and aimed to describe how forms were salient (or not) both within a community (in-group) as well as to out-group members and how this, then, could be linked with language change. According to Labov and Trudgill, features of which speakers are aware are *salient* variants and these can be classed as either *markers* or *stereotypes*. Variables which are non-salient in the speech community or to the individual speaker are called *indicators*. The difference between indicators and the other classifications is that indicators only display variation on the social level (i.e., among the different social classes) but not stylistic variation. Their status, however, can change over time. Markers, on the other hand, are salient but only to in-group members and display variation on both the social and stylistic levels (Labov calls this “consistent stylistic and social stratification,” 1994, p. 78). Markers are subject to change due to their salience (assuming that when a feature is salient it can be controlled which gives the speaker a choice when constructing utterances). Lastly, stereotypes are salient to both in-group and out-group members and often have an extra high level of awareness attached to them. However, due to their status as stereotype, they often function as a basis for negative comments and are often misrepresentations of vernacular speech. Stereotyped features, though, might enjoy widespread prestige among in-group speakers. This dual status of stereotyped features means that they not only are subject to correction and hypercorrection (Labov, 1994, p. 78) but also that they may not necessarily be likely to change, due to their ultra-salient status as this “may inhibit accommodation.” (Trudgill, 1986, p. 125).

According to Kerswill and Williams (2002), salience is “a notion which seems to lie at the cusp of language internal, external and extra-linguistic motivation […] which we can provisionally define rather simply as a property of a linguistic item or feature that makes it in some way perceptually and cognitively prominent.” (ibid.: 81). In their (2002) paper, Kerswill and Williams review several empirical studies of salience (including Trudgill, 1986) and conduct their own study investigating vowels, consonants and non-standard grammatical features in Milton Keynes, Reading and Hull. Based on their results and a discussion of the social embedding of forms, Kerswill and Williams conclude that it is not possible to set up any conditions which are either necessary or sufficient in order for a linguistic phenomenon to be salient and that the only prerequisite for salience seems to be that “its presence and absence must be noticeable in a psychoacoustic sense” (2002, p. 105). So “while language-internal factors play a part, it is in the end sociodemographic and other extra-linguistic factors that account for the salience of a particular feature” (ibid.: 81).

Branching out from pure sociolinguistic research, Hollmann and Siewierska (2006) take a socio-cognitive approach to salience. They agree with Kerswilll and Williams' emphasis on the importance of social factors but “see cognitive-perceptual factors as primary” (ibid.:209) because “linguistic items are will normally be more or less free from social values when they come into existence. It is only after they have emerged that social forces can start working on them” (ibid.). Thus, they place emphasis on cognitive-perceptual factors in determining salience as they see them as not only prior to any social factors but also as governing whether a form becomes subject to social evaluation.

In one of the more recent publications on salience within sociolinguistics (Rácz, 2013), we find a differentiation between cognitive (primary) and social (secondary) salience. Rácz' study is based in the area of sociophonetics and he sees salience as ultimately connected with surprisal. While related, cognitive salience is seen as separate from social salience and he defines the relationship between the two as follows: “Cognitive salience is an attribute of variation that allows language users to pick up on it, whereas social salience means that variation is already used to carry social indexation.” (ibid.: 37). This conceptualization of salience seems to support that presented by Hollmann and Siewierska (2006) above and brings in a useful distinction: that between the individual and the community level. It is clear that any consideration of the cognitive level must be concerned with individuals only, but also that individuals form communities, which allows us to extend our focus from the individual to the community. We return to this below in the conceptualization of language as a CAS.

### The enregisterment of social meaning

Rácz is not the only one to consider the role of social meaning in the study of salience. Honeybone and Watson (2013) in their study of Liverpool English phonology based on Contemporary, Humorous, Localized Dialect Literature suggest that a likely factor of the social salience of linguistic forms is the form's status as a local variant, indexing local identity. Similar results were also found for morphosyntactic and lexical forms in Tyneside English in Jensen (2013) who defines salience as the association of social content and linguistic forms in the cognitive domain. Thus, we see here that the social aspect is seen as crucial in the degree of salience of a number of non-standard forms.

Linked to the role of social meaning of local forms in speakers' identity constructions and often invoked in sociolinguistic studies as explanations of language variation and change are Silverstein's social indexicality (2003) and Agha's process of enregisterment (2003). Silverstein (2003, p. 217) directly maps his idea of different levels of social indexicality onto Labov's indicators, markers and stereotypes. Labov's *indicators*, Silversein argues, are forms used by all members of a particular social group and they thus index only the speakers' macro-social identity (ibid). *Markers*, on the other hand, are more intricate as they index not only macro-social identity but also style. He concludes on the topic of markers that “[w]hat Labov and followers have graphed in the so-called sociolinguistic marker is the dialectical process of indexical order for members of the standard-register informed language community as an articulated macro-social/micro-social fact” (ibid.: 220). Finally, Silverstein comments that *stereotypes* are markers whose interpretation is now wholly in the *n* + 1st order indexical field, i.e., the social connotations of the linguistic form are presupposed before the original (*n*-th order) interpretation (ibid.: 220). Connected to the notion of indexical order and the social indexicality of forms is enregisterment which describes “processes through which a linguistic repertoire becomes differentiable within a language as a socially recognized register of forms” (Agha, 2003, p. 231). Indeed, it can be argued that the (*n* + 1)+1st order indexical value of a linguistic form expresses the enregistered meaning of the form.

Johnstone (2009, p. 164), who investigates the indexicality of Pittsburghese, presents an overview of Silverstein's levels of indexicality and links them, very helpfully, with Agha's (2003) processes of enregisterment. We can summarize these in the following way:

- *n*th order indexicality/first order: this describes a linguistic form whose frequency of use patterns according to the socio-demographic background of the speakers (gender, class, region, age).
- *n*+1st order indexicality/second order: this describes a linguistic form which has acquired a social meaning which reflects dominant ideologies in the speech community (e.g., language correctness). At this stage, the form and social meaning are noticed by speakers.
- (*n*+1)+1st order indexicality/third order: this describes a linguistic form which has acquired an additional indexical meaning (in addition to its first order index) which results in it being interpreted in light of a different ideology (than the second order index). It is on this level that we find the additional layer of social value and where the form has been enregistered in the community. A link has been established between the use of the form and the social value (e.g., localness of the speaker).

As we can see, Silverstein's indexicality gives an account for how the social meaning of linguistic forms emerges on the level of the community and Agha's enregisterment describes the processes which cement the third order indexical values of these forms in the community.

But why do linguistic forms suddenly become enregistered in a community? Beal (2000; 2009) and Johnstone (2009, but most explicitly 2010) have argued that it is in times of change that the re-interpretation or resemiotization (that is, re-indexing of social meaning and enregisterment) of linguistic forms in terms of third order indexical meaning takes place. Johnstone's (2010) main argument is that in times of disruption (she focuses on globalization), very local forms come to index different social meanings. The features become the topic of conversation and they are used to differentiate members of different speech communities. However, most importantly, Johnstone argues that the idea of local speech as unique (and thus enregistered) solidifies the link between speech and place which renders other indexicalities (such as class, gender or age) less accessible.

If we acknowledge the cognitive aspect of salience (as is done e.g., by Jensen, 2013 and Rácz, 2013), then indexicality and enregisterment are useful aspects to consider. However, in processes of both indexicality and enregisterment, the attachment of social value to linguistic forms must take place on the level of the individual first and then spread to the community level from this point. Below, I bring in a psycholinguistic perspective as a way of unifying the social and cognitive aspects of language use.

### Social meaning in an exemplar framework

By viewing language as a CAS (Beckner et al., 2009), we can account both for the link between the social and the cognitive aspects of language via exemplar theory (on the level of the individual) as well as the link between the individual and the community-level patterns of enregistered social meaning.

According to Beckner et al (ibid.: 2), the key features of language as a CAS are:

- The system consists of multiple agents (the speakers in the speech community) interacting with one another.
- The system is adaptive; that is, speakers' behavior is based on their past interactions, and current and past interactions together feed forward into future behavior.
- A speaker's behavior is the consequence of competing factors ranging from perceptual mechanics to social motivations.
- The structures of language emerge from interrelated patterns of experience, social interaction, and cognitive processes.

What this means, then, is that speakers make choices about their own language (idiolect) but that these individual choices across a community result in emergent patterns of language use on a community level (ibid.: 14–15). Within this conceptualization of language, speakers' individual grammars are constructed as exemplar frameworks (ibid.:7).

Exemplar theory was first introduced in psychology in the 1970s as a model of perception and categorization and it has since then been adopted by linguistics and extended to the study of speech sounds and word recognition (Bybee, 2001, 2010; Pierrehumbert, 2001, 2003, 2006) among other areas. In short, exemplar models posit that “people represent categories by storing individual exemplars in memory, and classify objects on the basis of their similarity to these stored exemplars” (Nosofsky and Johansen, 2000, p. 375). Thus, exemplar theory presupposes richly detailed memory of exemplars, it is nonanalytic and works instead to match exemplars in a network fashion and it relies on probabilities and frequencies to do so (Mendoza-Denton, 2007; Barsalou, 2012; Fowler and Magnuson, 2012).

Pierrehumbert (2001) proposes that memories of tokens are stored in cognitive clouds where similar exemplars are stored close together and dissimilar ones far apart. The individual tokens or exemplars can be stored in several cognitive clouds depending on their categorization. In this way, the remembered tokens represent the range of variation encountered. A token can, for instance, be a word stored with information about particular acoustic features perceived (with phoneme-level exemplars stored separately, Drager, 2015, p. 154), the linguistic context in which it occurred and the social situation of when it was encountered (including formality levels and social information about the person who uttered it). If exemplars are frequently activated (either in production or perception), they remain at the forefront of the network “cloud” and are more easily activated again (they “carry the highest weight values,” Drager, 2015, p. 155). Both perception and production can be biased by the attachment of non-linguistic information to stored linguistic exemplars. In other words, social characteristics of interlocutors and the attitudes a speaker holds toward an interlocutor affect how we perceive their speech and how we address them (Niedzielski, 1999; Hay et al., 2006; Drager, 2015, p. 155–156).

According to Campbell-Kibler (2011), exemplar theory has appealed to linguistic theory generally, but the link between extralinguistic information and linguistic forms has been adopted and explored by sociolinguists and sociophoneticians in particular. She further states that “(e)xemplar theory's emphasis on the details of individual linguistic tokens makes it straightforward to link social information to extremely specific linguistic units and it is a compelling framework for further exploration of the linguistic character of sociolinguistic connections.” (ibid.: 437). And while an exhaustive survey of all studies exploring the attachment of social meaning to linguistic variables is impossible to undertake here (even if focusing only on studies which couch their interpretation of results in exemplar theoretical terms), I will here summarize a few which have been selected to show exemplar-based accounts pertaining to both production and perception as well as different linguistic levels.

Hay et al. (2006) investigated the effect of perceived speaker identity on the perception of near/square diphthongs which are currently merging in New Zealand English. Listeners were shown a photo of a speaker (older/younger, middle class/working class) and listened to a pre-recorded wordlist of unmerged near/square items. While the results of the study were quite complex, overall, listeners seemed to be influenced by the social characteristics displayed by the photos. When listeners thought they were listening to an older speaker (who would be likely to produce unmerged diphthongs), they performed more accurately on the word identification task than when they thought they were listening to a younger speaker (who would be more likely to use merged forms), even though the auditory input was the same. According to the authors, this indicates that listeners treat the words as being ambiguous (when the think they are produced by a younger speaker) as they expect the vowels to be merged to a greater extent. Their results for the manipulation of the speakers' social class were less clear, but listeners seemed to expect middle class speakers to be less merged than working class speakers (2006, p. 479). Hay, Warren and Drager suggest that these results support an exemplar-based model of speech perception where exemplars are linked to social characteristics.

More recent work by Drager (2015) investigates both perception and production of *like* among adolescents in a New Zealand all girls' school. She takes a qualitative, ethnographic approach to the investigation of identity construction among the different social groups in the school (all centered on the use or non-use of the school Common Room) but also employs quantitative acoustic analyses and experimental designs. Her variable, *like*, can have both grammatical (verb, adverb, noun, etc.) and discursive (discourse marker, quotative, approximative adverb, etc.) functions (ibid.: 76–77), and she investigates both grammatical and acoustic differences in the production, use and perception of this single lemma. I will just focus on her results for the production aspects here, where Drager found that the girls' use of phonetic variants was related to whether they used the school Common Room (and thus were part of the “normal” social groups) or not (and thus identified as “weird” and as different from the “normal” groups). She states that “this finding provides evidence that linguistic variables are correlated with a speaker's stance and that speakers actively adopt and reject linguistic variants as part of the construction of their identity.” (ibid.: 148).

Campbell-Kibler (2011) investigated the perception of variants of the variable (ING), -*in* and -*ing*, through a matched guise experiment which contained three guises: -*in*, -*ing*, and a neutral guise which contained no (ING) tokens. Her initial hypothesis was that listeners' expectations would be influenced by speakers' regional accent and that this would impact the perceptions of (ING). However, instead she found that the two variants were associated with different social features: -*ing* speakers were seen as more intelligent/educated and more articulate (than -*in* and neutral speakers) whereas -*in* speakers were perceived as being more informal and less likely to be gay (than -*ing* and neutral speakers). Thus, Campbell-Kibler concludes that “in some cases, variants of the same variable function independently as loci of indexically linked social meaning” (ibid.: 423).

Finally, also within sociolinguistic studies, both Rácz (2013) and Jensen (2013), who specifically investigate the topic of salience, suggest exemplar theory as a way of explaining the link between the social and the linguistic in the cognitive, and Foulkes and Docherty (2006) argue that an exemplar-based model of phonological knowledge offers the most productive means of modeling socio-phonetic variation as it offers a unified account of how socio-phonetic and linguistic material might be learned and stored. They conclude that “the interweaving of sociophonetic and linguistic information in speech is so complete that no natural human utterance can offer linguistic information without simultaneously indexing one or more social factor” (ibid.: 419). Indeed, Foulkes (2010) goes as far as stating that “[e]xemplar theory appears to be the most promising candidate to construct a cognitively-realistic, integrated theory of phonological knowledge, speech production, and speech perception in which indexical knowledge is not marginalized but central.” (ibid.: 32). We see that indexical knowledge, then, again appears and is deemed to be central to the organization of an exemplar network.

## Questionnaire study

This section reports on the variables under study (in Section Linguistic Variables), the design of the research instrument and the data yielded from the collection of questionnaires (Section Questionnaire Design and Output). The aim of the questionnaires was to investigate whether the local forms of the variables (do+NEG), (our), (told), (throw), and (go) are salient to Tyneside speakers and to investigate if participants' *affiliation* with Newcastle and the wider Tyneside area had any impact on their awareness and frequency ratings of speech containing Tyneside vernacular features.

### Linguistic variables

This section will briefly introduce the linguistic variables (the vernacular forms) studied here. While this section aims to introduce the variables to the reader, the main focus will be on how they can be formally described as well as how frequent they are. Further descriptions, including etymology, can be found elsewhere (e.g., Beal, 1993, 2004, 2010; Beal et al., 2012; Jensen, 2013, 2015).

As a way to gage the frequency of use of the different forms, a mini-corpus of Tyneside speech was compiled consisting of 24 dyadic interviews collected in Newcastle and Gateshead by local interviewers. The interviews selected were collected in the period 2007–2009 and are part of the Diachronic Electronic Corpus of Tyneside English^1^. More information about this corpus can also be found in Jensen (2013, 2015). The 48 speakers were distributed across social class, age and gender in the following way: 27 working class speakers and 29 middle class speakers, 29 young speakers (ages 17–34) and 27 older speakers (35+), 23 male speakers and 25 female speakers. The tokens were extracted using AntConc and included a variety of spellings^2^ for each variable, in order to find all tokens in the corpus.

The frequencies of forms are given here first and foremost to help readers unfamiliar with the variety. Secondly, the corpus frequencies given below are also compared to the perceived frequencies given in the questionnaire study in Section Analysis and Results of Frequency Judgments below. As such, this paper does not attempt to investigate links between actual frequencies and perceived frequencies or hypothesize on the role of relative or absolute frequencies of vernacular forms to their level of salience. Indeed, the topic of interest in this paper is the link between forms' perceived frequencies and salience.

(do + NEG)

Sentential negation with *do* in Tyneside English is realized as *divn't* (see examples below) and this form dominates the full present tense paradigm apart from the third person singular, which is *doesn't* (possibly realized as *dizn't*, see Rowe, 2007). The mini-corpus contained a total of 1663 tokens of sentential negation with *do;* 96 of these were in a vernacular form (5.8%).

1. Ah I just *divn't* want to get kidnapped. [07-08/N/ML/159]
2. The bars open late now divn-t they [07-08/N/RM/512]

(our)

The first person plural possessive pronoun in Tyneside English is *wor* and while this form is unique to the Tyneside area (Jensen, 2013), indeed the first person standard pronoun paradigm has been nearly completely re-organized in Tyneside English (this includes the use of *us* in both the plural subject and singular object, for instance). The mini-corpus contained 236 tokes of the first person plural possessive pronoun, 70 (29.7%) of which were *wor*.

(3) Me and Kerry have known each other like, all wor life [07-08/T/BB/929]
(4) Oh yeah, we're great friends with wor next door neighbors [07-08/N/VL/3892]

(told)

In Tyneside English, the past tense of the verb *to tell* is *telt*, which occurs both in the simple past as well as in constructions with the past participle. The compiled mini-corpus contained only 84 tokens of this variable out of which 5 (6%) were local forms.

(5) I telt O'Brien about them [07-08/N/ML/159]
(6) didn-t want to be telt what to do [07-08/N/PS/243]

(throw)

In Tyneside English we find a different lexical verb for the verb *to throw*, namely *to hoy*. This verb follows the regular paradigm and also occurs in the present participle (as *hoying*) and the past participle (*hoyed*). The corpus featured a total of 40 tokens with 11 (27.5%) being vernacular forms.

(7) they hoy it in the microwave and all [07-08/N/PM/85]
(8) the police used to hoy you over the wall so you'd get in free when you were little. [08-09/N/TS/556]

(go)

Finally, the verb *to go* is realized as *gan* in Tyneside English (present tense and present participle only) and is considered a separate verb (rather than a reflection of phonological differences between Standard English and Tyneside English; for more on this see Jensen, 2015). There is some variability in the vernacular paradigm as it seems to occur both with −*s* in all persons (as is common for some Northern verbs in the present tense, see Beal, 2010) and without (possibly following either the regular Standard paradigm or as subject to the Northern Subject Rule, Beal, 2010; Jensen, 2015). The mini-corpus featured a total of 2289 tokens of this variable; 202 (8.8%) of these were vernacular forms.

(9) Every-time you gan somewhere in that castle, shotgun shell! [07-08/G/DM/456]
(10) They constantly had me mam ganning up to the school to talk about us and stuff like that [07-08/N/PS/243]

### Questionnaire design and output

The questionnaire consisted of three separate tasks. Task one was a frequency judgment task which asks participants to indicate how frequent they believe certain forms are. Task 2 asked participants about their own language use and tested whether they can identify Tyneside features, and task 3 aimed to establish the participants' affiliation with the local area. The original questionnaire tested 12 different vernacular variables as well as four filler variables, but the part reported here will focus on only the five included in this paper (the full account can be found in Jensen, 2013).

The format of the questionnaire was inspired by Burbano-Elizondo (2008), who carried out a study of Sunderland English (another North Eastern British variant). In her study, she implemented an “affiliation”-score which she matched against informants' assessments of sentences featuring non-standard forms. She found a correlation between the informants' level of positivity toward Sunderland and their assessments of non-standard forms.

The section below gives further information about the general considerations of the questionnaire design including the counterbalancing scheme, the construction of example sentences and the use of filler sentences and controls overall. Section Analysis and Results of Frequency Judgments describes each task in more detail and includes information about the number of example sentences and fillers used and the type of output generated.

#### Overall questionnaire design

**Figure 1:**
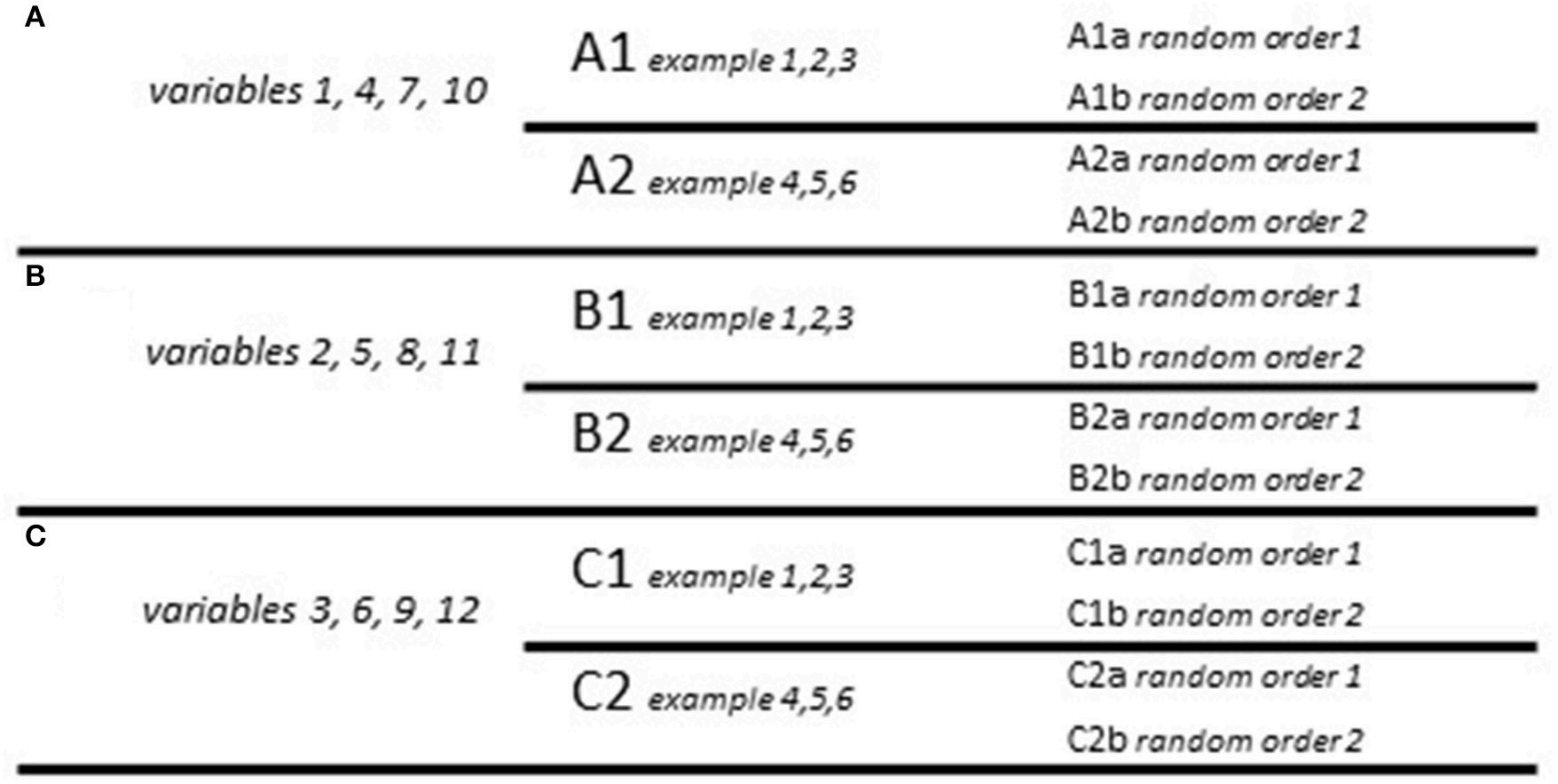
**Counterbalancing scheme**.

The questionnaire featured a brief introduction to its objectives and what participants were required to do. Each of the three tasks also featured a brief description of the task at hand and an example of how the participants should indicate their answers. Due to the high number of variables in the original questionnaire (12 vernacular variables + 4 filler variables), three overall versions of the questionnaire were created (A, B, C) each of which tested only four vernacular variables in task 1. For each version, two subversions were created which featured different example sentences containing the different variables (resulting in A1, A2, B1, B2, C1, C2). Finally, for each of these subversions 2 editions were created which featured the example sentences in random order (thus giving A1a, A1b, A2a, A2b, etc.). The tasks presented participants with both sentences containing Tyneside English forms, sentences containing standard forms and filler sentences containing either common non-standard forms (i.e., not local to the Tyneside area) or ungrammatical forms. The counterbalancing scheme can be found in Figure 1 mentioned below. Note that this is based on e example sentences in task 1.

The example sentences in tasks 1 and 2 were given in direct speech which formed part of small scenarios in order to make them more pragmatically acceptable (Schütze, 1996; Buchstaller and Corrigan, 2011; Buchstaller et al., 2013). This strategy also helps in making the written forms of the dialect variables less odd to the participants as they occur in the form of direct speech, and informants may then be more likely to judge them without prescriptivist influence. In addition, the example sentences used simple vocabulary (Cowart, 1997) in order to avoid sentences being rated negatively due to participants' unfamiliarity with the vocabulary used. The context in which the direct speech example sentences occurred was based on interactions between four fictional characters (John, Peter, Emily, and Betty) and described everyday set in everyday situations.

As mentioned above, the questionnaires also contained four filler variables, which functioned as control sentences in tasks 1+2 (in addition to the Standard English sentences). Fillers prevent participants from remembering and deliberating prior ratings and perhaps realizing what the underlying variable being tested is (Buchstaller and Corrigan, 2011). The fillers used took the form of two common non-standard forms (use of *ain't* and *they was*) and two ungrammatical forms (missing past tense inflection on verbs in combination with the adverb *yesterday* and erroneous use of the past tense form of an irregular main verb in negative sentences with *didn't*). Cowart (1997) also suggests that the fillers used represent different levels of unacceptability. In this study, the control sentences can be grouped on three levels of unacceptability. The standard forms of the vernacular sentences (which can be classed as a type of control too) would be expected to be rated as most frequent, as they are fully well-formed sentences. Participants would be expected to rate the common non-standard filler sentences as less frequent, as they are likely to be seen as less well-formed than the standard sentences but possible to some speakers. Finally, the ungrammatical filler sentences would be expected to be rated as most infrequent as they are likely to be completely non-acceptable to participants.

The example sentences used were all taken from either the DECTE corpus (for Tyneside English forms) or the BNC (for the fillers) and modified to fit the example context and edited for simplicity to avoid ratings based on structural complexity (Schütze, 1996). For the non-grammatical fillers, this meant actually making them ungrammatical and, for the Standard English forms, this meant converting the original Tyneside English form to the standard form.

#### Task structure and output

This section will provide further information about the structure of the individual tasks, what their aims are and what kind of output they yield.

##### Task 1

The aim of task 1 was to uncover how frequent participants believe certain forms to be. As mentioned above, there are three versions of the questionnaire (versions A, B, C) and task 1 tests four different variables on each of these versions (each variable is featured three times in order to increase reliability of ratings, Cowart, 1997). In total, task 1 featured 36 sentences (12 sentences in Tyneside English, 12 in Standard English and 12 fillers). Participants were asked to rate each sentence on a scale from 1 to 7. A rating of 1 was described as “This sentence is never used here” and a rating of 7 as “I hear this all the time. People use this a lot.” There were no verbal descriptions given to the ratings in between. A 4-point scale with verbal descriptors was used in Buchstaller et al. (2013), and while this is perhaps more appealing to participants (as it may be easier to identify with verbal descriptors as opposed to numbers) and it avoids a median value, the use of an interval scale allows for the use of parametric tests in the analysis phase. In addition to running the risk of being perceived as an ordinal scale (Cowart, 1997, p. 70–72), the use of verbal descriptors would also yield data unsuitable for parametric testing and thus non-parametric (i.e., less powerful) statistical methods would have to be used. The output of this task takes the form of numerical ratings from 1 to 7, which can then be averaged for each variable.

##### Task 2

The second task consisted of two parts: firstly, it aimed to establish how participants rate the frequency of their own use of particular forms and, secondly, if they can correctly identify local variants. The questionnaires tested all 12 variables in this task and included only the Tyneside English variants and the filler variables. This task featured 12 Tyneside English sentences (one for each variable) and 12 filler sentences (each of the four fillers occurred three times). Like task 1, task 2 also asked participants to use a 7-point scale to rate the example sentences. In this task, the verbal descriptors were 1: “I would never say this” and 7: “I say this all the time.” Due to prescriptivist pressure, participants were probably more likely to find this direct approach more invasive (compared to task 1), as they were asked to rate their own language. However, collecting both direct and indirect frequency judgments allows us to investigate how different variables are viewed in a community (Buchstaller and Corrigan, 2011). In the second part, participants were asked to indicate if the example sentences contained any local forms and to circle the word(s). This taps into their language awareness and requires that participants can be explicit about which features can be classified as belonging to the local area.

The output generated by this task is two-fold: the first output is similar to that of task 1, only this is a reflection of participants' own use (to the extent that they are able to gage it). This allows for comparisons between perceived “other” use and perceived “own” use with results telling us something about how forms are perceived in the community. The second output, the “awareness score,” describes participants' performance on the identification task and summarizes participants' answers to the two parts (first a yes/no question and, second, the identification itself). The “awareness score” is thus simply a numeric expression of the total number of correct identifications, i.e., a correct indication of YES in the first question and a correctly circled form in the second part of this task yielded a score of one. This score was calculated for each variable (the average number of correct identifications of this variable across participants) as well as for the participants as a group (the average number of correct identifications across all variables). The awareness scores tell us if participants are explicitly aware of local forms and connect them with the area.

##### Task 3

The third task measured participants' attitudes toward their local area, including the extent to which they feel an affiliation with the area. In this task, participants were asked to indicate to what extent they agreed with 10 statements which fell into five categories: opinion of the local area, orientation, network, self-definition, and attitude to dialect. These 10 statements also had to be rated on a scale from 1 to 7, where 1 was described as “I disagree strongly” and 7 as “I completely agree.” No verbal descriptions were given for the intermediate values. This section also featured background questions about participants' gender, age, education, socio-economic class, area where they grew up and if they had ever lived outside the Tyneside area.

The 10 statements and their categories were:

1. I am proud to be from Newcastle (opinion of Newcastle/Gateshead)
2. I'm more interested in local news than national or international news (orientation)
3. I consider myself to be a Geordie (self-definition)
4. I like the way people speak in Newcastle (attitude to dialect)
5. Most of my friends are from Newcastle (network)
6. I have a Geordie accent (self-definition)
7. I feel I have more in common with people from Newcastle (network)
8. I like the way I speak (attitude to dialect)
9. I'm more likely to strike up a conversation with a stranger if I know they are from Newcastle (orientation)
10. I like living in Newcastle (opinion of Newcastle/Gateshead)

The output generated by this task is a “local affiliation score” which was calculated as an average of participants' ratings of the 10 statements. This score can be compared to participants' performance on the other tasks in order to investigate whether a locally-rooted social identity is linked with perceived frequency of; perceived own use of; and identification of vernacular forms. It is this affiliation score which allows us to explore possible links between social identity and language perceptions.

As mentioned earlier, the composite affiliation score generated by the responses to this task is based on Burbano-Elizondo's work on Sunderland English. In her 2008 study, she employed a combination of different qualitative and quantitative methods in the construction of her *Index of Sunderland affiliation (ISA)* (2008, p. 126). While the present questionnaire study does not have a qualitative component, by incorporating questions about participants' orientation and opinion of the local vernacular in the local affiliation score, it aims to cover, in a quantitative manner, a similar range of topics.

#### Overview of collected data

Participants for the questionnaire study were recruited using the snowball method and, in total, 143 questionnaires were collected (summer of 2012). No particular social or age groups were targeted; the only criterion for participation was that participants identified themselves as Tyneside locals. The data was split into age groups after collection following the median of the participants' reported age (median age = 47), which also gave the best distribution across the other social categories (class and gender). Class is operationalized in terms of the informants' own definition of themselves (6 participants did not indicate class). The social stratification of the participants can be seen in Table 1 below.

**Table 1:** **Distribution of questionnaire participants based on social information**.

**WC**				**MC**			
**Younger (15–47)**		**Older (48**+**)**		**Younger (15–47)**		**Older (48**+**)**	
**Male**	**Female**	**Male**	**Female**	**Male**	**Female**	**Male**	**Female**
12	34	7	32	11	14	4	23

While this study will not further discuss the different behaviors of members of different social categories in detail, the above table provides the reader with an overview of the participants in the study. Overall, we can see that males were the hardest participants to reach, older males especially and middle class older males in particular. As a general observation, it should be added that middle class participants were harder to find when relying on people's own definition of themselves; however, many participants who identified as working class indicated high levels of education such as university degrees (see Jensen, 2013, in press for a discussion of this).

### Analysis and results

This section describes the collected questionnaire data and presents the different analyses and results based on the output described above.

#### Analysis and results of frequency judgments

**Figure 2:**
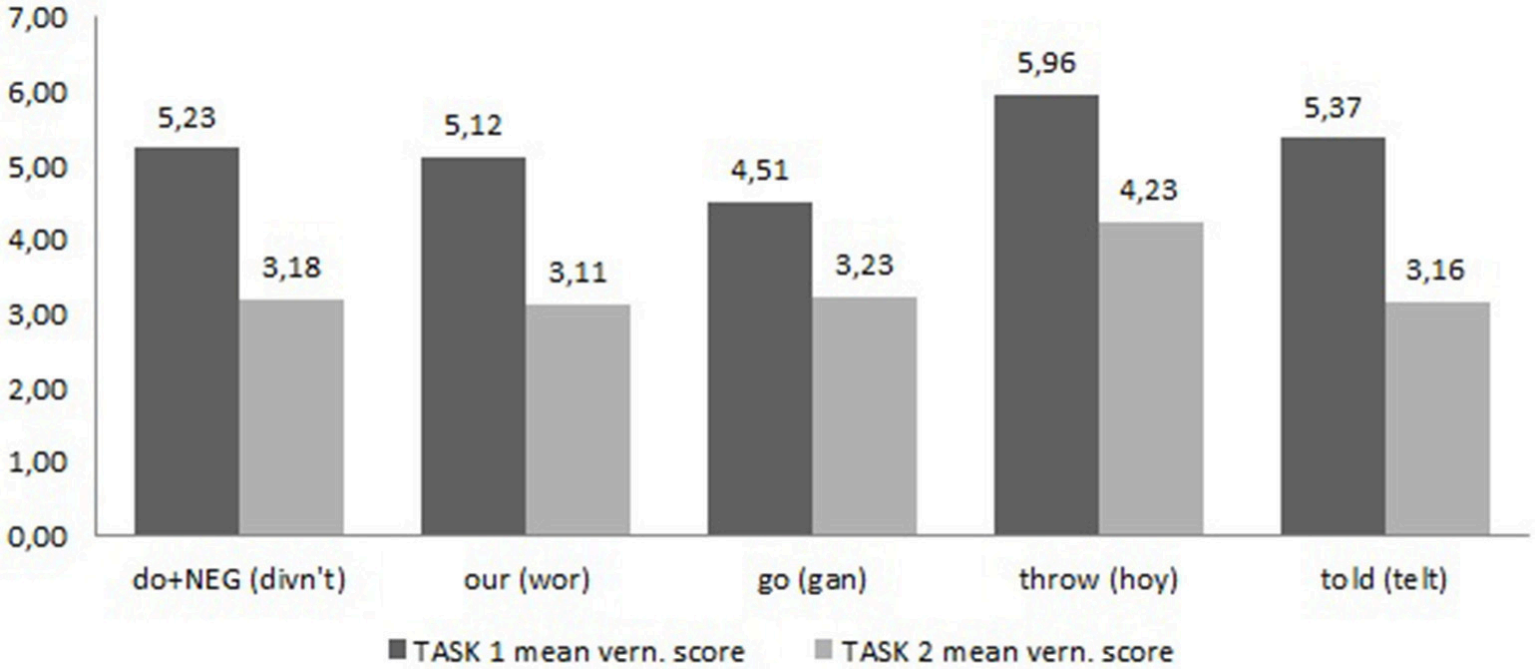
**Perceived frequency ratings compared**.

Comparing the ratings of the vernacular example sentences in tasks 1 and 2 gives us an indication of the status of the variables (see Figure 2). The reader should bear in mind that the ratings for task 1 are based on 46–49 responses as not all variables were included in each questionnaire version in task 1. The means for task 2 are based on 138–143 responses.

Dependent *t*-tests found significant differences between participant ratings for all variables and an overview of results is given in Table 2 below.

**Table 2:** *****T***-test analysis of mean vernacular scores for tasks 1 and 2**.

**Variables**	***N*s**	**TASK 1 mean**	**TASK 1 st.dev**	**TASK 2 mean**	**TASK 2 st.dev**	**Difference (1–2)**	***t***	***p***	**95% C.I**.	**Cohen's *d* (effect size)**
(do + neg)	45	5.20	1.51	3.38	2.34	1.82	5.521	0.000	1.2–2.5	0.92
(our)	46	5.12	1.29	3.80	2.26	1.32	4.203	0.000	0.7–1.9	0.72
(go)	45	4.54	1.76	3.27	2.32	1.27	3.947	0.000	0.6–1.9	0.62
(throw)	47	6.04	0.92	4.51	2.18	1.53	5.259	0.000	0.9–2.1	0.91
(told)	47	5.32	1.30	2.43	1.98	2.89	10.673	0.000	2.3–3.4	1.73

As we can see from the table, participants rate the use of vernacular forms by others as more frequent compared to their own use and significantly so. This indicates that participants are aware of the stigma surrounding non-standard forms. Interestingly, the perceived frequencies of forms do not match up particularly well with the actual frequencies from the mini-corpus. Across all variables, questionnaire participants generally overstate the use of the local forms. Table 3 below summarizes the frequencies from the corpus and also gives the corresponding means of tasks 1 and 2 from the questionnaire. In addition, the means from the questionnaires (which fall between 1 and 7) have been calculated into percentages (i.e., scores out of 100) to ease the comparison.

**Table 3:** **Corpus frequencies**.

**Variables**	***N***	**Vernacular forms N/%**	**TASK 1 mean/%**	**TASK 2 mean/%**
do + NEG (*divn't*)	1663	96/5.8	5.20/74.29	3.38/48.29
(*wor* for *our*)	236	70/29.7	5.12/73.14	3.80/54.29
go (*gan*)	2289	202/8.8	4.54/64.86	3.27/46.71
throw (*hoy*)	40	11/27.5	6.04/86.29	4.51/64.43
told (*telt*)	84	5/6	5.32/76	2.43/34.71

Correlational tests (Pearson product-moment) showed large positive correlations between the corpus frequencies and both task means, however, the results are not significant with an alpha level of 0.05. Task 1: *r* = 0.475, *n* = 5, *p* = 0.419 with a shared variance of 22.6%. Task 2: *r* = 0.801, *n* = 5, *p* = 0.103, 64.2% shared variance.

#### Analysis and results of identification task

The output of this task was two “awareness scores”; one for the participants and one for the individual variables. Overall, participants were good at correctly identifying the Tyneside forms with a mean score of 9.08 (*N* = 143, standard deviation = 2.55, minimum = 0, maximum = 12). With regards to the individual variables, we can see from Table 4 below that all five variables were identified over 90% of the time.

**Table 4:** **Identification of vernacular forms**.

**Variables**	***N***	**Mean**	**Correct identification**	**Correct identification in %**
do + NEG (*divn't*)	143	0.93	133	93
(*wor* for *our*)	143	0.91	130	91
go (*gan*)	143	0.91	130	91
throw (*hoy*)	143	0.94	134	94
told (*telt*)	143	0.94	134	94

The awareness scores of the variables capture the degree to which participants were aware of them and connected them with the local area. In that way, they tell us something about the salience of the variables as participants have to be aware of the forms and link them to the area in order to be able to identify them.

#### Analysis and results of affiliation task

As outlined above, the tasks consisted of 10 statements (in five categories) and participants had to indicate the extent to which they agree by using a 7-point scale. Table 5 below shows participants' ratings of the different categories. We can see that they have a generally positive opinion of their local area, that they generally identify as *Geordies*, and that they have a favorable opinion of the local variety. Finally, while they have local networks, their orientation is not focused on the local area.

**Table 5:** **Affiliation ratings**.

**Task 3**	***N*s**	**Minimum**	**Maximum**	**Mean**	**Standard deviation**
Opinion	143	1.0	7.0	6.11	1.23
Self-definition	143	1.0	7.0	5.46	1.38
Attitude	143	1.0	7.0	5.17	1.46
Network	143	1.0	7.0	4.67	1.51
Orientation	143	1.0	7.0	3.44	1.54
Scores across all five categories	143	1.0	7.0	4.97	1.00

Before exploring the correlations between participants' affiliation score and their performance on the other tasks, a principal components analysis (Oblimin/oblique rotation) was carried out in order to test if the affiliation score can actually be perceived as a composite index at all. A PCA works by reducing data and revealing underling structures in larges sets of variables. Here, it was used to investigate the extent to which the categories in the “affiliation index” cluster together, i.e., the extent of their association (Pallant, 2007, p. 179) and thus the extent to which they can be seen as parts of a composite score.

The data passed the initial suitability assessment (Kaiser-Meyer-Oklin value = 0.774, Bartlett's Test of Sphericity = *p* < 0.000). The coefficients of the correlation matrix were mainly above 0.3 and a high positive correlation (*r* = 0.520) between the categories “attitude” and “opinion” was found, clearly linking these two categories. The PCA of the five categories showed the presence of only one component with an eigenvalue exceeding 1.0 (2.548) explaining 50.962% of the variance as we see from Table 6 below.

**Table 6:** **Components found in principal component analysis of the five categories**.

**Component**	**Initial eigenvalues**			**Extraction sums of squared loadings**		
	**Total**	**% of Variance**	**Cumulative %**	**Total**	**% of Variance**	**Cumulative %**
1	2.548	50.962	50.962	2.548	50.962	50.962
2	0.803	16.064	67.026			
3	0.673	13.468	80.493			
4	0.541	10.821	91.315			
5	0.434	8.685	100.000			

**Figure 3:**
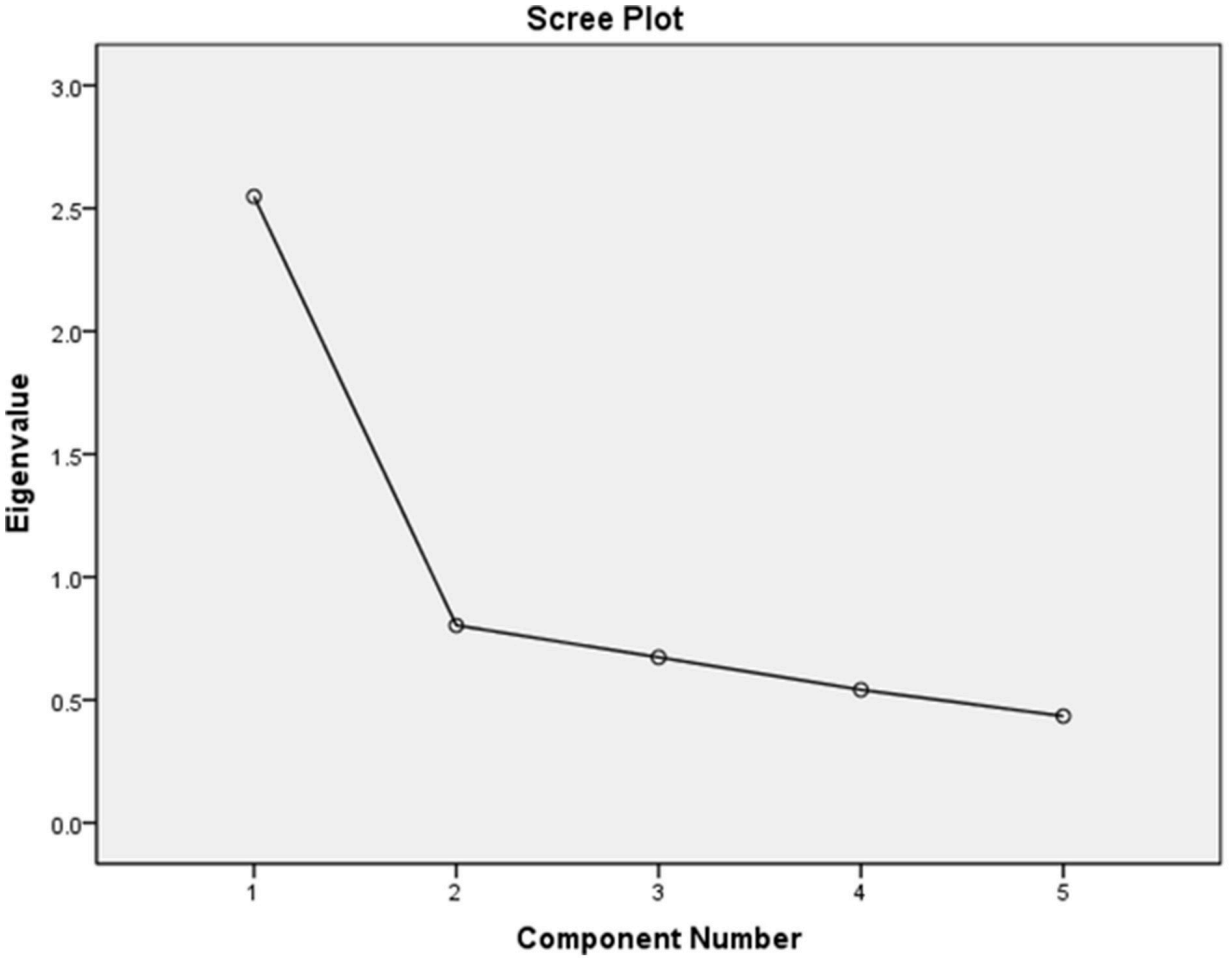
**Screeplot**.

This was further supported by the screeplot which showed a clear break after the first component, shown here in Figure 3.

The component matrix showed that all variables loaded strongly on this single factor (over 0.4). The factor weights indicate that “attitude” loads most strongly (and is thus the most important in the composite score) with a score of 0.764, followed by “opinion” (0.751), “network” (0.749), “self-definition” (0.697), and finally “orientation” (0.595.). Because only one component was found, rotation could not be performed. On the basis of this analysis, we can accept the affiliation score as a composite index.

The affiliation score was correlated (using Pearson's Product-Moment Correlation) with the ratings in task 1 (perceived frequency of other people's use) and task 2 part 1 (perceived frequency of own use). Table 7 below gives the correlations between participants' affiliation score and their ratings in the two tasks, respectively. Variability in the mean values of task 3 (affiliation index) and the *N*-values is due to missing answers in either task 1 or task 2 as variables with missing responses were excluded from the analysis.

**Table 7:** **Correlations: frequencies and local affiliation**.

	***N***	**Task 1**	**Task 3**	***r***	***p***	***N***	**Task 2**	**Task 3**	***r***	***p***
(do + NEG)	46	5.22	4.94	0.46	0.001	142	3.18	4.96	0.41	0.000
(our)	48	5.11	5.14	0.44	0.002	141	3.11	4.96	0.31	0.000
(go)	46	4.51	4.94	0.24	0.115	142	3.23	4.97	0.34	0.000
(throw)	48	5.96	5.15	0.48	0.001	141	4.23	4.97	0.36	0.000
(told)	49	5.37	4.82	0.13	0.382	140	3.16	4.96	0.33	0.000

For all variables, we see that the correlation between the ratings and the affiliation index is positive, i.e., the higher the affiliation score, the higher the rating of the vernacular forms. The most important result here is the *r*-value as that describes the level of correlation between the two scores. Usually, a value above 0.3 is interpreted as a medium value (which will be the threshold used here). While it is important that the *p*-value is low (below 0.05 to indicate a significant and reliable result), the value itself does not indicate the importance of the *r*-value (Dancey and Reidy, 2011, p. 188, Pallant, 2007, p. 132–33). In the table, cells which feature an *r*-value above 0.3 and a *p*-value below 0.05 have been shaded. We can see that there are significant correlations between the ratings for all variables in task 2 (participants' own use) and participants' affiliation scores and for three out of five variables in task one (frequency in other's use) and the affiliation index scores. In short, the more attached participants feel to the local area, the higher they rate both other people's use of vernacular forms but in particular their own. This indicates that local affiliation may influence perceptions of both other people's language use but also of own language use. This will be discussed further in Section Discussion and Conclusion below.

Finally, another Pearson test was run to see if there was any correlation between participants' affiliation score and their ability to correctly identify the vernacular variables. This was calculated on the basis of the responses to the individual variables (i.e., it was a point-biserial correlation with a bivariate variable, either correct or incorrect identification of the variable, and a continuous variable, the participants' affiliation score). As the identification task is a dichotomous variable, the mean values indicated are simply the mean of the coding, where 1 represented a correct identification and 0 an incorrect identification (either an erroneous identification or simply a missing answer). Again, cells with significant results (*p* < 0.05 and *r* > 0.3) have been shaded.

Table 8 above shows that, for three of the five variables, there is a significant correlation between participants' ability to correctly identify vernacular forms and the expression of local affiliation (as measured in the affiliation index). While none of the tests returned correlations above 0.3, we can see that (throw) came the closest with 0.220 (and also showed a highly significant correlation with *p* = 0.008) followed by (our), *r* = 0.203, *p* = 0.015). We can interpret these results as meaning that, at least for some vernacular features, there may be a tendency for level of local affiliation to positively impact explicit awareness of local vernacular forms.

**Table 8:** **Correlations: identification and local affiliation**.

	***N***	**Identification**	**Affiliation**	***r***	***p***
(do + NEG)	143	0.93	4.97	0.092	0.277
(our)	143	0.91	4.97	0.203	0.015
(go)	143	0.91	4.97	0.178	0.034
(throw)	143	0.94	4.97	0.220	0.008
(told)	143	0.94	4.97	0.075	0.377

## Discussion and conclusion

To summarize the above section, we saw that there was a difference in how speakers rate their own speech vs. that of others. The questionnaire participants rated all five variables as more frequently occurring on the speech of others than in their own to a significant degree. Furthermore, we saw that participants were very competent in identifying the five vernacular variables (all identified correctly over in 90% of occurrences) and connected them with the Tyneside area. The affiliation index allowed comparisons between participants' performance in the different tasks with a composite measure of their attachment to the local area. While not conclusive across all five variables, these comparisons showed that there may be a connection between speakers' affiliation with their local area and their awareness of the use of local features, in particular in their perceptions of the extent to which they themselves use local forms.

We can see, then, that the variables investigated here seem to be enregistered as unique to Tyneside (cf. Johnstone, 2010; Honeybone and Watson, 2013). Their status as indices of Tyneside local identity can become strengthened over time with use and increased exposure. In this way, we can see speakers as active participants in the construal of the social meaning of linguistic forms. From an exemplar theoretical perspective, we can argue that this enregistered status affects their storage in the exemplar network cloud. If unique local forms are stored as separate entries (rather than exemplars of standard forms), they are perhaps in a better position to be imbued with social value. This would also mean that they escape the pressure of prescriptive rules which face non-standard forms otherwise. This would presuppose, however, that the speakers perceive the vernacular forms as being unique to Tyneside, something which the results reported here indicate is the case. We can perhaps then also suggest there is a close link between salience and social value and that they are important factors in a model of language meaning, with unique forms (or forms perceived to be unique, rather) being the best carriers of social meaning as they are more positively viewed in the community and not stigmatized to the same extent as non-unique forms (Jensen, 2013). This link between the social value of the form and the linguistic form itself is what we can capture by the term salience if salience is defined as the association of social content and linguistic forms in the cognitive domain.

As mentioned in Section The Enregisterment of Social Meaning, it has been suggested that processes of enregisterment are set in motion by disruption in some form. In the case of the Tyneside area, this catalyst could be the transformation which the area has seen over the last several years. A hundred years ago, the Tyneside area was an area defined by heavy industry (such as shipbuilding) and the town of Newcastle was the retail center for the whole of the north of England. When the heavy industry began to wane in the mid-1900s, Newcastle strengthened its position as a retail center. More recently, focus has shifted to the consumption of culture with both a modern art gallery and an all-glass concert hall as well as several bars and pubs lining the banks of the river Tyne. Finally, Newcastle is also a popular student city and has the fifth largest student population in England and Wales (Beal et al., 2012; Jensen, 2013). It can thus be argued that this transformation of the Tyneside conurbation which the Tyneside speakers have witnessed (but which has not influenced the stereotypical associations held by out-group members, see Watt, 2002) provides the optimal conditions for enregisterment processes of certain local forms to happen.

An additional aspect of the social value argument is that attachment of meaning to particular local forms (in this case localness) allows forms to parti cipate in the stylization of social personae (Podesva, 2011a,b; Drager, 2015, p. 157). Drager (2015, p. 157–163) gives a step-by-step account of how the construction of a social persona through the adoption and non-adoption of different features (linguistic and otherwise) may be “understood within an exemplar-based hybrid model.” (ibid.: 157). In short, both the presence and absence of different features are part of creating a social persona, that is, different features can index different personae to different extents and sometimes it is the combination of variants over a range of variables which delimit one persona from another. Not all features which could become parts of a social persona do, however, and speakers are still influenced by social convergence and divergence (Giles and Powesland, 1975) and they are free to shift their personae over the course of an interaction.

In the study presented here, consideration of speakers' creation of social personae (which in this case are centered around signaling localness) may explain the full correlation between all variables in task 2 part 1 and the affiliation score; speakers with a high affiliation score also want to present themselves as “true Geordies” (which can be done by claiming to use features perceived to be local to a large extent). This presupposes, of course, that participants can identify the local features in the examples sentences (task 2 part 2) and thus that they are aware of them. As we saw from the results of the identification task, all variables in this study were correctly identified as local over 90% of the time. Not only do participants' ratings then indicate that they are aware of which features are local, but also that an awareness of what being a Geordie might entail and how to enact it. Additionally, the adoption of a Geordie persona also indicates a positive attitude both toward Geordie as an identity (and with that the local area) but also about showing it. This suggestion is backed up by findings reported in Beal (1999) and Jensen (2013). Indeed, Beal (1999:45) states that “[p]erhaps the preservation of stereotypical pronunciations in key words like “Toon,” along with the leveling toward supraregional rather than national norms reported by Watt (2002), represent a strategy for maintaining the positive aspects of the “Geordie” stereotype: friendliness and a strong sense of regional identity, whilst dissociating oneself from the negative, “grim up north” aspects of that stereotype.”

Finally, it should be self-evident that language exists on two levels; the individual level and the community level. We saw in Section Social Meaning in an Exemplar Framework how CAS theory suggests that speakers make choices about their own language but that these individual choices result in emergent patterns of language across a community. Similarly, we can also see language, or, rather, meaning, as operating on two levels; the first is the denotational level (which captures the communicative meaning of the speech signal) and the second is the sociolinguistic meaning, which is tied to speakers' linguistic identities. If we see speakers' individual grammar as constructed as exemplar frameworks, then the merger of these two levels of meaning is unproblematic. This is also supported by the literature reviewed in Section Social Meaning in an Exemplar Framework. As for the local Tyneside variables investigated here, we can thus see them as carrying heavy indexes of “locality” within the individuals' exemplar clouds and that this will affect the way speakers and listeners use and perceive the forms. On the community level, this will then result in different patterns of use across groups and across time. I will leave it up to future studies to investigate how these patterns might emerge and develop.

## Author contributions

The author confirms being the sole contributor of this work and approved it for publication.

### Conflict of interest statement

The author declares that the research was conducted in the absence of any commercial or financial relationships that could be construed as a potential conflict of interest.

## Footnotes

^1^http://research.ncl.ac.uk/decte/.

^2^This was necessary for two reasons: first to collect all morphological forms of the words (e.g., *hoy, hoyed*, and *hoying*) but, two, also because there is some variability in the transcription conventions used within the corpus (so *divn't* may be found as *divn't, divvent, divn-t*).

## References

[B1] Agha A. (2003). The social life of cultural value. Lang. Commun. 23, 213–273. 10.1016/S0271-5309(03)00012-0

[B2] Barsalou L. W. (2012). The human conceptual system, in The Cambridge Handbook of Psycholinguistics, eds Spivey M. Joanisse M. McRae K. (New York, NY: Cambridge University Press), 239–258.

[B3] Beal J. (1993). The grammar of Tyneside and Northumbrian English, in Real English: The Grammar of English Dialects in the British Isles, eds Milroy J. Milroy L. (Harlow: Longman), 187–213.

[B4] Beal J. (1999). Geordie Nation: Language and identity in the North-east of England. Lore Lang. 17l, 33–48. Available online at: http://collections.mun.ca/PDFs/lorelang/LoreandLanguageVol17No01021999.pdf

[B5] Beal J. (2000). From Geordie Ridley to Viz: popular literature in Tyneside English. Lang. Lit. 9, 343–359. 10.1177/096394700000900403

[B6] Beal J. (2004). English dialects in the North of England: morphology and syntax, in A Handbook of Varieties of English, Vol. 2, eds Kortmann B. Schneider E. W. Burridge K. Meshtrie R. Upton C. (Berlin: Mouton de Gruyter), 114–141.

[B7] Beal J. (2009). Enregisterment, commodification, and historical context: “Geordie” versus “Sheffieldish”. Am. Speech 84, 138–156. 10.1215/00031283-2009-012

[B8] Beal J. (2010). An Introduction to Regional Englishes. Edinburgh: Edinburgh University Press.

[B9] Beal J. L. Burbano-Elizondo C. Llamas C. (2012). Urban North-Eastern English: Tyneside to Teesside. Edinburgh: Edinburgh University Press.

[B10] Beckner C. Blythe R. Bybee J. Christiansen H. M. Croft W. Ellis C. N. (2009). Language is a complex adaptive system: poition paper. Lang. Learn. 59(Suppl. 1), 1–26. 10.1111/j.1467-9922.2009.00533.x

[B11] Buchstaller I. Corrigan K. P. (2011). Judge not lest ye be judged: exploring methods for the collection of socio-syntactic data, in Language Variation - European Perspectives III, eds Gregersen F. Parrott J. K. Quist P. (Amsterdam: John Benjamins), 149–160.

[B12] Buchstaller I. Corrigan K. P. Holmberg A. Honeybone P. Maguire W. (2013). T-to-R and the Northern Subject Rule: questionnaire-based spatial, social and structural linguistics. English Lang. Linguist. 17, 85–128. 10.1017/S1360674312000330

[B13] Burbano-Elizondo L. (2008). Language Variation and Identity in Sunderland. Ph.D. thesis, Sheffield University, Sheffield.

[B14] Bybee J. (2001). Phonology and Language Use. Cambridge: Cambridge University Press.

[B15] Bybee J. (2010). Language, Usage and Cognition. Cambridge: Cambridge University Press.

[B16] Campbell-Kibler K. (2011). The sociolinguistic variant as a carrier of social meaning. Lang. Var. Change 22, 423–441. 10.1017/S0954394510000177

[B17] Cowart W. (1997). Experimental Syntax: Applying Objective Methods to Sentence Judgments. Thousand Oaks, CA: Sage Publications.

[B18] Dancey C. P. Reidy J. (2011). Statistics Without Maths For Psychology, 5th Edn. Harlow: Pearson Education.

[B19] Drager K. (2015). Linguistic Variation, Identity Construction and Cognition. Berlin: Language Science Press.

[B20] Foulkes P. (2010). Exploring social-indexical knowledge: a long past but a short history. J. Lab. Phon. 1, 5–39. 10.1515/labphon.2010.003

[B21] Foulkes P. Docherty J. G. (2006). The social life of phonetics and phonology. J. Phon. 34, 409–438. 10.1016/j.wocn.2005.08.002

[B22] Fowler C. A. Magnuson J. S. (2012). Speech perception, in The Cambridge Handbook of Psycholinguistics, eds Spivey M. Joanisse M. McRae K. (New York, NY: Cambridge University Press), 3–25.

[B23] Giles H. Powesland P. F. (1975). Speech Style and Social Evaluation. London: Academic Press.

[B24] Hay J. Warren P. Drager K. (2006). Factors influencing speech perception in the context of a merger-in-progress. J. Phon. 34, 458–484. 10.1016/j.wocn.2005.10.001

[B25] Hollmann W. B. Siewierska A. (2006). Corpora and (the need for) other methods in a study of Lancashire dialect. Zeitschrift für Anglistik und Amerikanistik 54, 203–216. 10.1515/zaa-2006-0210

[B26] Honeybone P. Watson K. (2013). Salience and the sociolinguistics of Scouse spelling: exploring the contemporary, humourous, localised dialect literature of Liverpool. English World Wide 34, 305–340. 10.1075/eww.34.3.03hon

[B27] Jensen M. M. (2013). Salience in Language Change: A Socio-Cognitive Study of Tyneside English. Ph.D. thesis, University of Northumbria, Newcastle.

[B28] Jensen M. M. (2015). Two directions of change in one corpus: phonology vs morphosyntax in Tyneside English. Globe 1, 43–71. 10.5278/ojs.globe.v1i0.698

[B29] Jensen M. M. (in press). Education, class vernacular awareness on Tyneside, in Perspectives on Northern British Englishes, eds Hancil S. Beal J. (Berlin: De Gruyter).

[B30] Johnstone B. (2009). Pittsburghese shirts: commodification and the enregisterment of an urban dialect. Am. Speech 84, 157–175. 10.1215/00031283-2009-013

[B31] Johnstone B. (2010). Indexing the local, in Handbook of Language and Globalization, ed N. Coupland (Chichester: Wiley-Blackwell), 386–404.

[B32] Johnstone B. Andrus J. Danielson A. E. (2006). Mobility, indexicality, and the enregisterment of “Pittsburghese.” J. English Linguist. 34, 77–104. 10.1177/0075424206290692

[B33] Johnstone B. Kiesling F. S. (2008). Indexicality and experience: exploring the meanings of /aw/-monophthongization. J. Sociolinguist. 12, 5–33. 10.1111/j.1467-9841.2008.00351.x

[B34] Kerswill P. Williams A. (2002). “Salience” as an explanatory factor in language change: evidence from dialect levelling in urban England, in Language Change: The Interplay of Internal, External and Extra-Linguistic Factors, eds Jones M. C. Esch E. (Berlin: Mouton de Gruyter), 81–110.

[B35] Labov W. (1972). Sociolinguistic Patterns. Philadelphia: University of Pennsylvania Press.

[B36] Labov W. (1994). Principles of Linguistic Change. Volume one: Internal Factors. Oxford: Blackwell Publishers.

[B37] Mendoza-Denton N. (2007). Socio-linguistic extemnsions of exemplar theory: comments on Flege, Khattab, and Darcy, Peperkamp and Dupoux, in Laboratory Phonetics 9, eds Cole J. Hualde J. I. (Berlin: Mouton de Gruyter), 443–453.

[B38] Niedzielski N. (1999). The effect of social information on the perception of sociolinguistic variables. J. Lang. Soc. Psychol. 18, 62–85. 10.1177/0261927X99018001005

[B39] Nosofsky R. M. Johansen M. K. (2000). Exemplar-based accounts of “multiple-system” phenomena in perceptual categorization. Psychon. Bull. Rev. 7, 375–402. Available online at: http://www.cogs.indiana.edu/nosofsky/pubs/2000_rmn-mkj_pbr_Exemplar.pdf 11082849

[B40] Pallant J. (2007). SPSS Survival Manual, 3th Edn. Maidenhead: Open University Press, McGraw-Hill Education.

[B41] Pierrehumbert J. B. (2001). Exemplar dynamics: word frequency, lenition and contrast, in Frequency Effects and the Emergence of Linguistic Structure, eds Bybee J. L. Hopper P. J. (Amsterdam: John Benjamins), 137–157.

[B42] Pierrehumbert J. B. (2003). Probabilistic phonology: discrimination and robustness, in Probabilistic Linguistics, eds Bod R. Hay J. Jannedy S. (Cambridge, MA: The MIT Press), 177–228.

[B43] Pierrehumbert J. B. (2006). The next toolkit. J. Phon. 34, 516–530. 10.1016/j.wocn.2006.06.00326944793

[B44] Podesva R. J. (2011a). Salience and the social meaning of declarative contours: three case studies of gay professionals. J. English Linguist. 39, 233–264. 10.1177/0075424211405161

[B45] Podesva R. J. (2011b). The California vowel shift and gay identity. Am. Speech 86, 32–51. 10.1215/00031283-1277501

[B46] Rácz P. (2013). Topics in English Linguistics [TiEL]: Salience in Sociolinguistics: A Quantitative Approach. Berlin: Walter de Gruyter.

[B47] Rowe C. (2007). He divn't gan tiv a college ti di that, man! A study of do (and to) in Tyneside English. Lang. Sci. 29, 360–371. 10.1016/j.langsci.2006.12.013

[B48] Schütze C. T. (1996). The Empirical Base of Linguistics: Grammaticality Judgments and Linguistic Methodology. Chicago: University of Chicago Press.

[B49] Silverstein M. (2003). Indexical order and the dialectics of sociolinguistic life. Lang. Commun. 23, 193–229. 10.1016/S0271-5309(03)00013-2

[B50] Trudgill P. (1986). Dialects in Contact. Oxford: Blackwell Publishers.

[B51] Watt D. (2002). I don't speak with a Geordie accent, I speak, like, the Northern accent”: contact-induced levelling in the Tyneside vowel system. J. Sociolinguist. 6, 44–63. 10.1111/1467-9481.00176

